# The Role of Neuregulin-1 in Steatotic and Non-Steatotic Liver Transplantation from Brain-Dead Donors

**DOI:** 10.3390/biomedicines10050978

**Published:** 2022-04-23

**Authors:** Marc Micó-Carnero, Araní Casillas-Ramírez, Alfredo Sánchez-González, Carlos Rojano-Alfonso, Carmen Peralta

**Affiliations:** 1 Institut d’Investigacions Biomèdiques August Pi i Sunyer (IDIBAPS), 08036 Barcelona, Spain; mico@clinic.cat (M.M.-C.); rojano@clinic.cat (C.R.-A.); 2 Hospital Regional de Alta Especialidad de Ciudad Victoria “Bicentenario 2010”, Ciudad Victoria 87087, Mexico; aranyc@yahoo.com (A.C.-R.); asg_4@live.com (A.S.-G.); 3 Facultad de Medicina e Ingeniería en Sistemas Computacionales de Matamoros, Universidad Autónoma de Tamaulipas, Matamoros 87300, Mexico

**Keywords:** neuregulin-1, brain death, liver transplantation, steatotic liver grafts, ischemia-reperfusion

## Abstract

Background. Brain death (BD) and steatosis are key risk factors to predict adverse post-transplant outcomes. We investigated the role of Neuregulin-1 (NRG1) in rat steatotic and non-steatotic liver transplantation (LT) from brain death donors (DBD). Methods: NRG1 pathways were characterized after surgery. Results: NRG1 and p21-activated kinase 1 (PAK1) levels increased in steatotic and non-steatotic grafts from DBDs. The abolishment of NRG1 effects reduced PAK1. When the effect of either NRG1 nor PAK1 was inhibited, injury and regenerative failure were exacerbated. The benefits of the NRG-1-PAK1 axis in liver grafts from DBDs were associated with increased vascular endothelial growth factor-A (VEGFA) and insulin growth factor-1 (IGF1) levels, respectively. Indeed, VEGFA administration in non-steatotic livers and IGF1 treatment in steatotic grafts prevented damage and regenerative failure resulting from the inhibition of either NRG1 or PAK-1 activity in each type of liver. Exogenous NRG1 induced greater injury than BD induction. Conclusions: This study indicates the benefits of endogenous NRG1 in liver grafts from DBDs and underscores the specificity of the NRG1 signaling pathway depending on the type of liver: NRG1-PAK1-VEGFA in non-steatotic livers and NRG1-PAK1-IGF1 in steatotic livers. Exogenous NRG1 is not an appropriate strategy to apply to liver grafts from DBD.

## 1. Introduction

Currently, 80% of grafts are obtained from donors after brain death (DBDs). However, brain death (BD) markedly decreases liver graft tolerance to preservation/reperfusion injury, as well as graft survival [1,2]. In clinical liver transplantation (LT), the shortage of hepatic graft donors, and consequently the increase in transplant waiting lists, has led centers to expand their organ acceptance criteria to marginal donors, such as steatotic liver grafts. Up to 50% of deceased donor livers are estimated to be steatotic, and steatosis is recognized as a key donor variable in the prediction of adverse post-transplant outcomes because hepatic steatosis implies a greater risk of organ dysfunction and primary non-function than transplants with non-steatotic livers [3,4]. The prevalence of steatosis is constantly increasing in society, so many steatotic livers, especially those with severe fatty infiltration, are discarded, thus exacerbating the critical shortage of liver donors [5]. Progress in therapeutic strategies aimed at reducing the inherent risk of dysfunction or failure that steatotic livers suffer after LT from DBDs is urgently required and would also help to more rapidly decrease the LT waiting lists. Neuregulin-1 (NRG1) is a neurotrophic factor that is highly expressed in the nervous system [6]. Experimental studies indicate that plasma NRG1 levels are reduced in nervous system disorders and neurodegenerative diseases [7,8]. In our view, the possibility that BD causes deficiencies in systemic brain-derived NRG1 levels and subsequent deficiencies in hepatic NRG1 levels that could adversely affect liver grafts from DBDs should not be dismissed. Interestingly, the liver is an important target for NRG1 [9]. Previous reports support the idea that NRG1 has diverse effects on hepatocytes. It has been evidenced that NRG1 exerts important metabolic effects in the liver since NRG1 improves insulin resistance in rats by activating ErbB3 and promoting the phosphorylation of AKT in hepatocytes [10], which leads to suppression of gluconeogenic gene expression and hepatic glucose output, in both lean and obese mouse livers [9]. Moreover, recently NRG1 has been demonstrated via the same mechanism of ErbB3-AKT activation, decreased TG levels, and inhibited steatosis in a hepatic cellular model of nonalcoholic fatty liver disease, thus validating a protective role of NRG1 in the pathogenesis of hepatic steatosis [10]. In addition to these metabolic benefits, in vitro cell viability studies have indicated that NRG1 has a direct cytoprotective effect against pentachlorophenol-induced toxicity in hepatocytes. In this sense, NRG1-β inhibited apoptosis by suppressing the activation of caspase-3 and attenuated inflammation, the expression of stress-related genes, cell cycle arrest, and DNA damage [11].

NRG1 has been shown to participate in the regulation of p21-activated kinase 1 (PAK1) [12,13,14]. PAK1 is expressed in the liver, and its activation inhibits cell death [15]; its over-expression also protects against inflammatory bowel diseases [16]. To our knowledge, no study has evaluated the potential benefits of the NRG1-PAK1 pathway in LT from DBDs. In addition, it is also well-known that PAK1 plays an important role in controlling vascular endothelial growth factor-A (VEGFA) levels [17]. Interestingly, PAK1 and VEGFA expression are positively regulated by NRG1 in different cell lines [18,19]. Several results have been reported on the beneficial role of VEGFA in the setting of hepatic surgery associated with warm ischemia, hepatic resections, and LT [20,21]. Therefore, a relationship between NRG1-PAK1-VEGFA in LT from DBDs should be not ignored.

Another growth factor could also be involved in NRG1 and PAK1 effects. The interaction between NRG1 and insulin growth factor-1 (IGF1) has been previously reported in rat cardiomyocytes, and a relationship between PAK1 and IGF1 has been demonstrated in cultured endothelial cells [22,23]. IGF1 is considered one of the main growth factors with beneficial properties in hepatic ischemia-reperfusion injury. In this sense, the administration of IGF1 protected the liver from warm ischemia-reperfusion injury [24] and improved the postoperative outcomes after LT [25]. Thus, in addition to a possible role of VEFGA on the effects of NRG1 and PAK1, the involvement of IGF1 in the signaling mechanisms of the NRG1-PAK1 pathway could also be considered in the setting of LT from DBDs.

This study aimed to evaluate the role of NRG1 in steatotic and non-steatotic LT from DBDs and to investigate whether PAK1 is possibly involved in the effects of NRG1 in these surgical conditions. We also assessed whether the potential NRG1-PAK1 signaling pathway can regulate VEGFA and/or IGF1 in steatotic and non-steatotic LT from DBDs. The idea is to elucidate the as yet unidentified pathophysiological mechanisms in steatotic and non-steatotic LT from DBDs and identify the mediator with beneficial effects that could be considered a therapeutic target to generate therapies in LT, or in contrast, mediators with deleterious effects that render its modulation inappropriate when protecting LT from DBDs.

## 2. Materials and Methods

### 2.1. Experimental Animals

This study was performed using male homozygous (obese [Ob]) and heterozygous (lean [Ln]) Zucker rats (Iffa Credo, L’Arbresele, France), 12 to 14 weeks of age. Zucker rats constitute a well-characterized model of genetic obesity. Homozygous Zucker rats (Ob) lack the cerebral leptin receptor and develop obesity at the age of 8 weeks because of markedly increased food intake and decreased energy expenditure. In contrast, heterozygous Zucker rats (Ln) have cerebral leptin receptors and maintain a lean phenotype throughout life. In addition, we used male Wistar rats (200–220 g) (Iffa Credo, L’Arbresele, France) fed with a choline-deficient or standard chow diet for 10 days as a nutritional obesity experimental model. Ob Zucker and Ob Wistar rats show severe macrovesicular and microvesicular fatty infiltration in hepatocytes (>60% steatosis) [21,26]. All procedures were conducted according to European Union regulations (Directive 86/609 EEC) for animal experiments.

### 2.2. Experimental Design

1. **LT** (*n* = 24 rats)*:* In subgroup 1.1 (*n* = 12 rats, 6 transplantations with steatotic grafts, 6 donor Ob Zucker rats, and 6 recipient Zucker Ln rats), Ob Zucker rats were anesthetized, ventilated, and maintained normotensively with a saline infusion for 6 h. Steatotic livers were subsequently flushed with University of Wisconsin (UW) solution, isolated, preserved in ice-cold UW solution for 4 h, and implanted into Ln Zucker rats. In subgroup 1.2 (*n* = 12 rats, 6 transplantations with non-steatotic grafts, 6 donor Ln rats, and 6 recipient Ln rats), the same surgical procedure was repeated but with Ln Zucker rats as donors and recipients [26,27,28]
2. **BD+LT** (*n* = 24 rats): In subgroup 2.1 (*n* = 12 rats, 6 transplantations with steatotic grafts, 6 donor Ob Zucker rats and 6 recipient Zucker Ln rats), Ob Zucker rats were anesthetized and ventilated. BD induction was performed with a frontolateral trepanation in rats, and a balloon catheter was introduced in the extradural space. The intracranial pressure was increased by inflating the balloon for one minute, which induced rapid brain injury and led to immediate BD [29,30]. Rats were maintained normotensively with a colloid infusion for 6 h. Next, livers were flushed with UW solution, isolated, preserved in ice-cold UW solution for 4 h, and implanted into Ln Zucker rats [26,27]. In subgroup 2.2 (*n* = 12 rats, 6 transplantations with non-steatotic grafts, 6 donor Ln rats, and 6 recipient Ln rats), the same BD surgical procedure and liver transplantation were repeated but with Ln Zucker rats as donors and recipients.
3. **BD+anti-NRG1+LT** (*n* = 24 rats): as in group 2 but with the administration of an antibody against NRG1 (10 mg/kg, i.v.) [31,32] immediately after BD induction.
4. **BD+PAK1 inhibitor+LT** (*n* = 24 rats): as in group 2 but with the administration of a highly selective inhibitor of PAK1 (IPA-3 (4 mg/kg, i.v.) [33]) immediately after BD induction.
5. **BD+anti-NRG1+IGF1+LT** (*n* = 24 rats): as in group 2 but with the administration of an antibody against NRG1 (10 mg/kg, i.v.) [31,32] and with IGF1 (400 μg/kg i.v.) [24], immediately after BD induction.
6. **BD+PAK1 inhibitor+IGF1+LT** (*n* = 12 rats): as in group 2.1 but with the administration of a highly selective inhibitor of PAK1 (IPA-3 (4 mg/kg, i.v.) [33]) and with IGF1 (400 μg/kg i.v.) [24], immediately after BD induction.
7. **BD+anti-NRG1+VEGFA+LT** (*n* = 24 rats): as in group 2 but with the administration of an antibody against NRG1 (10 mg/Kg, i.v.) and with VEGFA (10 µg/kg, i.v.) [34,35], immediately after BD induction.
8. **BD+PAK1 inhibitor+VEGFA+LT** (*n* = 12 rats): as in group 2.2 but with the administration of a highly selective inhibitor of PAK1 (IPA-3 (4 mg/kg, i.v.) [33]) and with VEGFA (10 µg/kg, i.v.) [34,35], immediately after BD induction.
9. **BD+NRG1+LT**. This group is divided into two subgroups, as follows. **(9.1) BD+NRG1(a)+LT** (*n* = 24 rats): as in group 2 but treated with NRG1 at a dose of 200 µg/kg, i.v. [36] immediately after BD induction; **(9.2) BD+NRG1(b)+LT** (*n* = 24 rats): as in group 2 but treated with NRG1 at a dose of 400 µg/kg, i.v. [36] immediately after BD induction.

Samples were collected 4 h after LT. The study conditions, including the doses and pre-treatment points used for the different drugs, were selected on the basis of the previous studies mentioned above and preliminary studies from our group. A cold ischemic period of 4 h is sufficiently long to induce liver injury after transplantation in both liver grafts while allowing high survival 4 h after transplantation. Thus, the experimental conditions used in this study were the most appropriate to evaluate the effect of NRG1 on injury and cell proliferation, as well as the NRG1 signaling pathways in steatotic and non-steatotic LT from DBDs.

To investigate the origin of NRG1, the following experimental groups were performed:

10. **Sham** (*n* = 12 rats, 6 Ob and 6 Ln Zucker rats): animals were submitted only to anesthesia and laparotomy.
11. **Prior to LT** (*n* = 12 rats, 6 Ob and 6 Ln Zucker rats): animals were anesthetized, and livers were subsequently flushed with University of Wisconsin (UW) solution, isolated, and maintained in cold ischemia in UW solution for 4 h.
12. **BD** (*n* = 12 rats, 6 Ob and 6 Ln Zucker rats): animals were anesthetized and ventilated. After BD induction, rats were maintained normotensively with colloid infusion for 6 h. In this experimental group, samples were collected at 10 min, 1, 2, and 6 h after BD induction.
13. **Prior to BD+LT** (*n* = 12 rats, 6 Ob, and 6 Ln Zucker rats): animals were anesthetized and ventilated. After BD induction, rats were maintained normotensively with colloid infusion for 6 h. Then, livers were flushed with University of Wisconsin (UW) solution, isolated, and maintained in cold ischemia in UW solution for 4 h.

To evaluate the effects of NRG1 in LT from DBDs in the nutritionally induced obesity experimental model, we performed the same surgical procedures under the same conditions as those described for groups 1–9 but using Wistar rats fed the choline-deficient diet (Ob Wistar rats) instead of Ob Zucker rats, and Wistar rats fed the standard chow diet (Ln Wistar rats) instead of Ln Zucker rats.

### 2.3. Biochemical Determinations

Plasma transaminases (alanine aminotransferase (ALT) and aspartate aminotransferase (AST)) were measured using standard procedures. Alkaline phosphatase (ALP), total bilirubin, Von Willebrand factor (vWF), and hyaluronic acid (HA) were determined in plasma using colorimetric (ALP: ab287823 from Abcam, Cambridge, UK) or immunosorbent (Total Bilirubin: MB730053 from MyBioSource, Inc., San Diego, CA, USA; vWF: CSB-E08438r from Cusabio, Wuhan, China; HA: DHYAL0 from R&D Systems, Minneapolis, MN, USA) commercial assay kits. NRG1 levels were determined in plasma and liver using an immunoassay kit (E-EL-R0790 from Elabscience Biotechnology Co., Ltd., Wuhan, China). PAK1, IGF1, VEGFA, hepatocyte growth factor (HGF), and transforming growth factor-β (TGF-β) levels were quantified in liver tissue with immunoassay kits (PAK1: MBS2887909 from MyBioSource, Inc., San Diego, CA, USA; and IGF1 (E-EL-R3001), VEGFA (E-EL-R2603), HGF (E-EL-R0496), and TGF-β (E-EL-R0084) from Elabscience Biotechnology Co., Ltd., Wuhan, China), according to the manufacturer’s instructions.

Lipid peroxidation was determined by measuring the formation of malondialdehyde (MDA) with the thiobarbiturate reaction [37]. Myeloperoxidase (MPO), an index of neutrophil accumulation, was measured photometrically with 3,3′,5,5′-tetramethylbenzidine as a substrate [37]. Hepatic edema was determined as follows: after resection, hepatic samples were weighed and then placed in a drying oven at 55 °C until a constant weight was obtained. In this determination, hepatic edema is represented by an increase in the wet-to-dry weight ratio [38].

### 2.4. Histology

To appraise the severity of hepatic injury, paraffin-embedded liver sections were stained with H&E and blind histological scoring was performed by a board-certified pathologist, using a point-counting method on an ordinal scale: grade 0, minimal or no evidence of injury; grade 1, mild injury consisting of cytoplasmic vacuolation and focal nuclear pyknosis; grade 2, moderate to severe injury with extensive nuclear pyknosis, cytoplasmic hypereosinophilia, and loss of intercellular borders; grade 3, severe necrosis with disintegration of hepatic cords, hemorrhage, and neutrophil infiltration; and grade 4, very severe necrosis with disintegration of hepatic cords, hemorrhaging, and neutrophil infiltration [37]. Liver steatosis was evaluated via red oil staining [39,40].

### 2.5. Immunohistochemistry

Liver samples were immunostained with a monoclonal antibody against proliferating cell nuclear antigen (PCNA) (DAKO, Agilent Technologies, Santa Clara, CA, USA). Samples were developed with diaminobenzidine and counterstained with hematoxylin. The proliferation index of PCNA-stained biopsy specimens was determined in 30 high-power fields. Data were expressed as the percentage of PCNA-stained hepatocytes per total number of hepatocytes [41]. The slides were blinded to the examiners, who had extensive experience in the evaluation of liver pathology.

### 2.6. Statistics

The statistical significance of different variables was determined via the non-parametric Kruskal–Wallis test. The Mann–Whitney U test was applied to groups showing significant differences, and adjusted *p*-values were calculated using the False Discovery Rate (FDR) method (*p* < 0.05 was considered significant).

## 3. Results

### 3.1. Role of NRG1 in Steatotic and Non-Steatotic LT from DBDs in a Genetic Obesity Experimental Model

**Figure 1:**
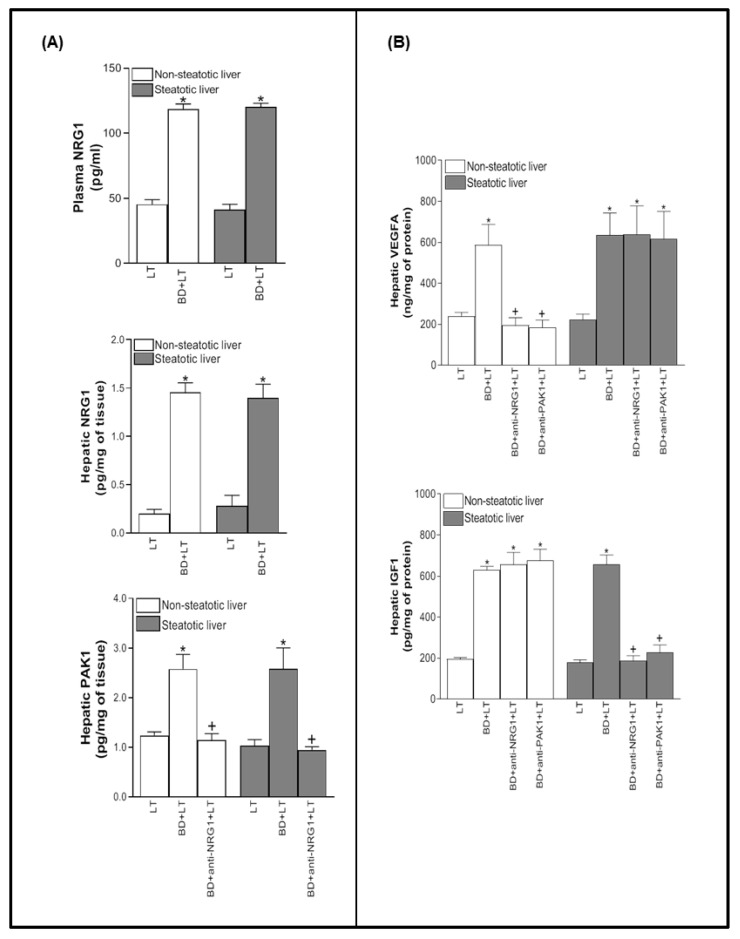
This is a figure. Schemes follow. Effect of NRG1 on PAK1, IGF1, and VEGFA levels in steatotic and non-steatotic LT from DBDs in a genetic obesity model. (**A**) Levels of NRG1 in plasma and levels of NRG1 and PAK1 in liver tissue. (**B**) Levels of IGF1 and VEGFA in liver tissue. * *p* < 0.05 vs. LT; ^+^ *p* < 0.05 vs. BD+LT.

Since plasma NRG1 levels are decreased in some central nervous system pathologies and the liver is one of the targets of NRG1 [7,9], we initially evaluated whether plasma NRG1 levels were associated with hepatic NRG1 levels in LT from DBDs. Our results indicated an increase in protein levels of NRG1 in plasma and liver grafts in the BD+LT group when compared with the results in the LT groups (Figure 1A).

NRG1 has been shown to participate in the regulation of PAK1 in cells and tissues different to the liver’s [12,13,14]. No study has yet evaluated the potential relationship of the NRG1 and PAK1 in the setting of liver transplantation from DBDs. We set out to investigate whether such a relationship was present in our experimental conditions in order to elucidate possible signaling pathways to explain the effects of NRG1. In this sense, we evaluated hepatic NRG1 levels in an experimental group of liver transplantations from DBDs (BD+LT group). As is shown in Figure 1A, our results indicated an increase in protein levels of NRG1 in liver grafts in the BD+LT group when compared with the results in the liver transplantation groups from donors that had not undergone BD (LT group). After that, we determined the levels of PAK1 in steatotic and non-steatotic liver transplantations from DBDs. Our results indicated an increase in protein levels of PAK-1 in steatotic and non-steatotic grafts in the BD+LT group when compared with the results in the LT group (Figure 1A). Therefore, both NRG1 and PAK1 mediators were increased in liver transplantation from DBDs. One way to find out if PAK1 is related to NRG1 is to inhibit the effect of NRG1 and evaluate the levels of PAK1 in this experimental condition. When we administered an antibody against NRG1 to inhibit the action of this mediator (BD+anti-NRG1+LT), decreased PAK1 levels were observed in both types of liver grafts (Figure 1A). This led us to conclude that the inhibition of NRG1 modifies the levels of PAK1 and observe that both mediators are related and that NRG1 regulates PAK1.

Next, we investigated whether the NGR1-PAK1 pathway was able to affect the IGF1 and VEGFA levels in steatotic and non-steatotic LT from DBDs. The inhibition of either NRG1 or PAK1 (BD+anti-NRG1+LT and BD+anti-PAK1+LT groups) decreased VEGFA in non-steatotic livers, while no changes were observed in the presence of steatosis when compared with the results obtained in the BD+LT group. On the other hand, in the presence of steatosis, the abolishment of NRG1 or PAK1 effects affected IGF1 since in steatotic livers from the BD+anti-NRG1+LT and BD+anti-PAK1+LT groups, IGF1 levels were lower than in the BD+LT group. In non-steatotic livers, IGF1 levels were similar in the BD+anti-NRG1+LT, BD+anti-PAK1+LT, and BD+LT groups (Figure 1B).

**Figure 2:**
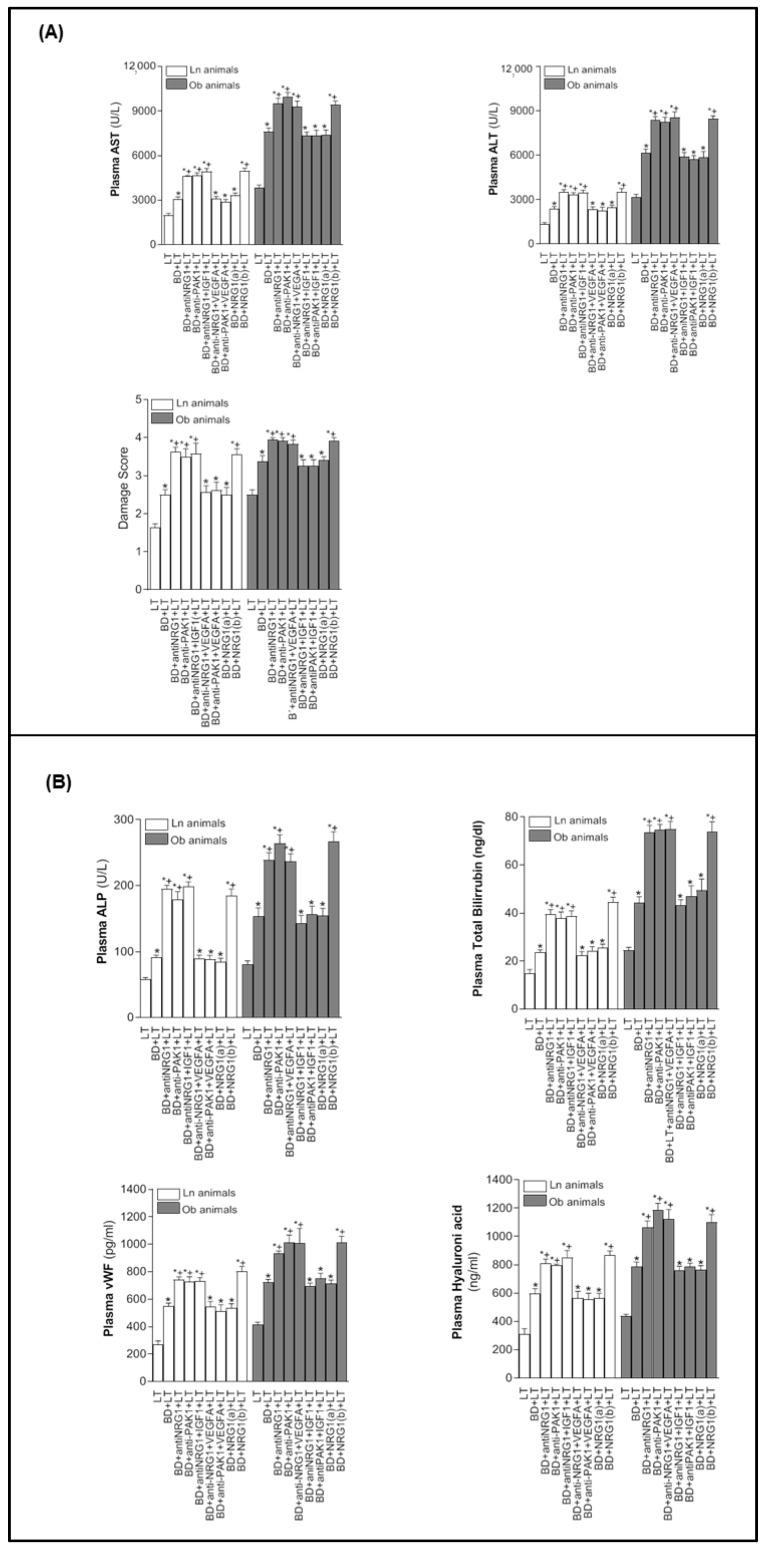
The relevance of NRG1 on injury in steatotic and non-steatotic LT from DBDs in a genetic obesity model. (**A**). ALT and AST levels in plasma, and damage score in the liver. (**B**) ALP, total bilirubin levels, vWF, and HA levels in plasma. * *p* < 0.05 vs. LT; ^+^ *p* < 0.05 vs. BD+LT.

**Figure 3:**
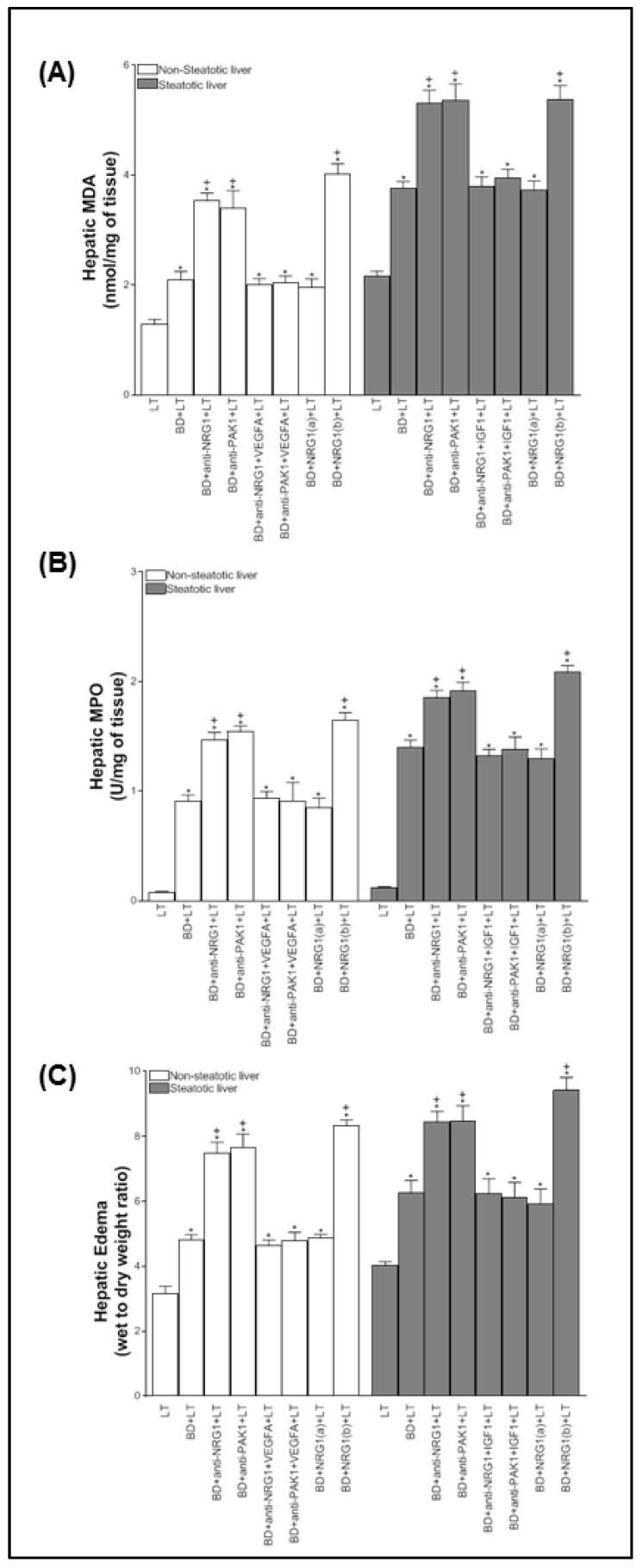
The relevance of NRG1 on inflammation in steatotic and non-steatotic LT from DBDs in a genetic obesity model. (**A**) MDA levels in liver tissue as an index of oxidative stress; (**B**) MPO levels in liver tissue as a parameter of neutrophil accumulation and (**C**) hepatic edema. * *p* < 0.05 vs. LT; ^+^ *p* < 0.05 vs. BD+LT.

**Figure 4:**
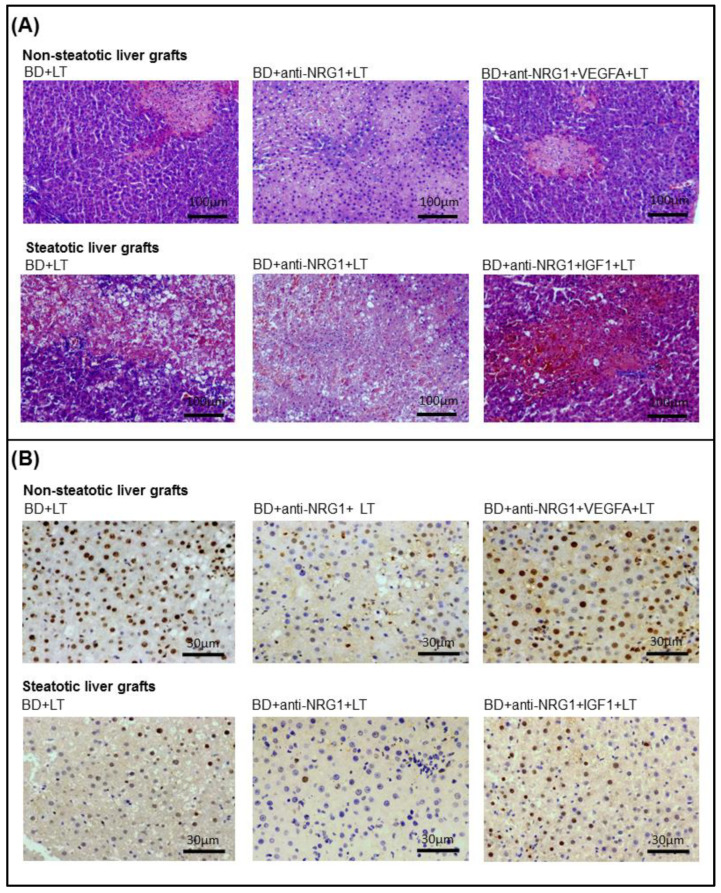
Representative photographs of histological changes and immunohistochemical staining of PCNA-positive cells in steatotic and non-steatotic LT from DBDs in a genetic obesity model. (**A**) Histological changes. In the BD+LT group, the histological evaluation of non-steatotic livers showed moderate multifocal areas of coagulative necrosis and neutrophil infiltration, randomly distributed throughout the parenchyma; severe, extensive, and confluent areas of coagulative necrosis were observed in the BD+anti-NRG1+LT group. When recombinant VEGFA was administered to the BD+anti-NRG1+LT group, liver tissue injury decreased, resulting in similar histological findings to those described in the BD+LT group. Steatotic liver grafts from BD+LT and BD+anti-NRG1+IGF1+LT showed extensive and confluent areas of coagulative necrosis, whereas the extent and the number of necrotic areas increased in the BD+anti-NRG1+LT group (4×). (**B**) Immunohistochemical staining of PCNA-positive cells. Non-steatotic liver grafts in the BD+anti-NRG1+LT group showed fewer positive cells than in the BD+LT and BD+anti-NRG1+VEGFA+LT groups. In steatotic liver grafts, fewer positive cells were observed in the BD+anti-NRG1+LT group in comparison with those in the BD+LT and BD+anti-NRG1+IGF1+LT experimental groups (20×).

**Figure 5:**
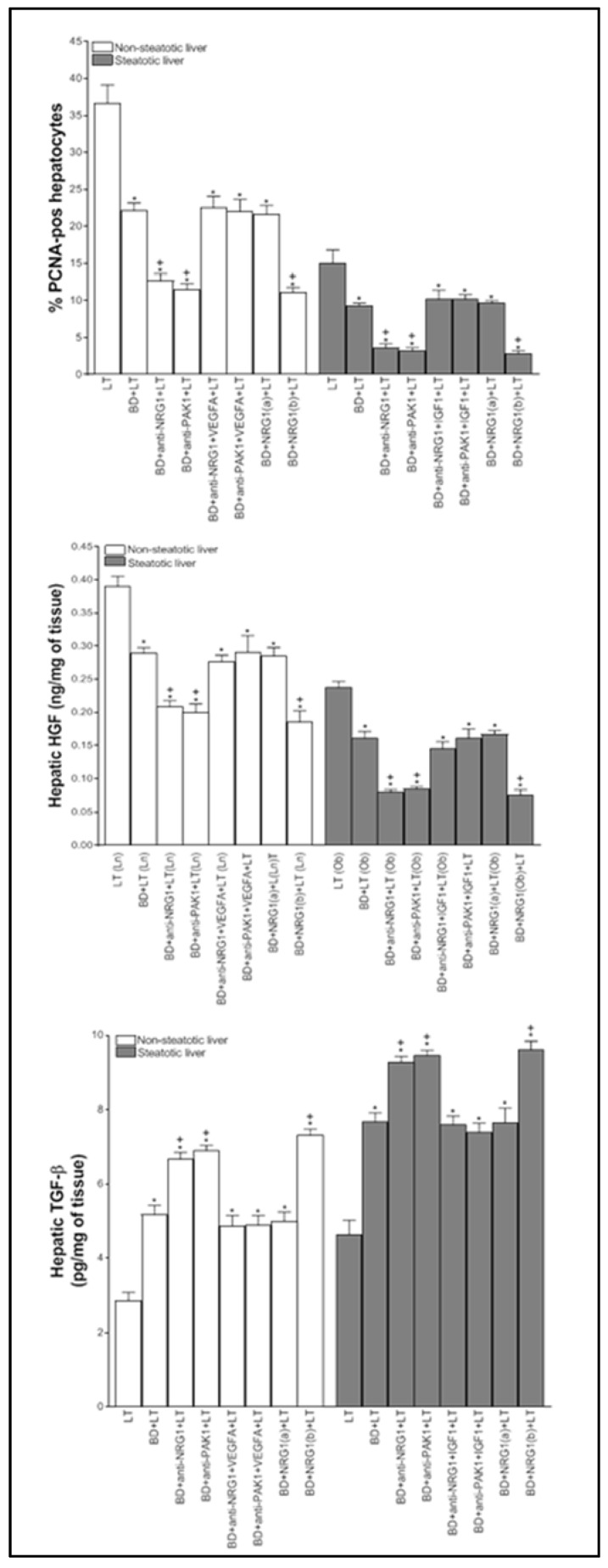
The relevance of NRG1 on regenerative failure in steatotic and non-steatotic LT from DBDs in a genetic obesity model. PCNA-positive-hepatocytes in liver grafts, HGF, and TGFβ levels in liver tissue. * *p* < 0.05 vs. LT; ^+^ *p* < 0.05 vs. BD+LT.

Next, the relevance of NRG1-PAK1-VEGFA and NRG1-PAK1-IGF1 on hepatic injury and proliferation in non-steatotic and steatotic LT from DBDs was investigated. The inhibition of either NRG1 or PAK1 activity (BD+anti-NRG1+LT and BD+anti-PAK1+LT groups) exacerbated hepatic injury since it increased transaminases, damage score values (Figure 2A), ALP, total bilirubin, and endothelial cell damage measured by vWF and HA levels (Figure 2B), in comparison with the BD+LT group, and in both liver types. This was associated with an exacerbated inflammatory response, as shown by the increase in oxidative stress as determined by MDA, neutrophil accumulation measured with MPO, and edema formation in non-steatotic and steatotic grafts of the BD+anti-NRG1+LT and BD+anti-PAK1+LT groups in comparison with the BD+LT group (Figure 3). In the BD+LT group, the histological evaluation of non-steatotic livers showed moderate multifocal areas of coagulative necrosis and neutrophil infiltration, randomly distributed throughout the parenchyma, while severe, extensive, and confluent areas of coagulative necrosis were observed in the BD+anti-NRG1+LT group (Figure 4A). In steatotic grafts of the BD+LT, severe, extensive, and confluent areas of coagulative necrosis were observed, whereas the extent and the number of necrotic areas were increased in the BD+anti-NRG1+LT group (Figure 4A). In terms of liver regeneration, BD+anti-NRG1+LT and BD+anti-PAK1+LT groups showed lower values of PCNA-positive hepatocytes in non-steatotic and steatotic grafts compared with the BD+LT group (Figure 4B and Figure 5). This reduction in proliferative cells was also associated with lower levels of HGF and higher levels of TGF-β in the BD+anti-NRG1+LT and BD+anti-PAK1+LT groups with respect to the BD+LT group, in both types of livers (Figure 5). It is well-known that HGF is a potent mitogen [42], and TGF-β is considered the principal inhibitor of hepatocellular proliferation [43].

Since these results reflecting exacerbated injury and regenerative failure in the BD+anti-NRG1+LT and BD+anti-PAK1+LT groups were associated with decreased VGFA and IGF1 in non-steatotic and steatotic livers, respectively, we then investigated whether endogenous NRG1-PAK1 exerted its benefits through VEGFA/IGF1. We administered VEGFA to rats with non-steatotic livers after pharmacological inhibition of the NRG1-PAK1 pathway (BD+anti-NRG1+VEGFA+LT and BD+anti-PAK1+VEGFA+LT groups), as well as exogenous IGF1 to those with steatotic livers after pharmacological inhibition of the NRG1-PAK1 pathway (BD+anti-NRG1+IGF1+LT and BD+anti-PAK1+IGF1+LT groups). In these conditions, the parameters of hepatic injury (transaminases, damage score, ALP, total bilirubin, vWF, HA, and the extent and number of necrotic areas, Figure 2 and Figure 4), inflammation (MDA, MPO, and edema, Figure 3) were decreased and liver regeneration (the PCNA-positive hepatocytes, HGF and TGF-β, Figure 4B and Figure 5) was ameliorated, in comparison with experimental groups in which the effects of NRG1 or PAK1 were inhibited (BD+anti-NRG1+LT and BD+anti-PAK1+LT groups), thus resulting in similar parameters to those in the BD+LT group with steatotic and non-steatotic livers. As control experiments, we treated non-steatotic livers undergoing the abolishment of NRG1 with IGF1 (BD+anti-NRG1+IGF1+LT) and administered VEGFA in steatotic livers in which NRG1 was blocked (BD+anti-NRG1+VEGFA+LT). As expected, the parameters of hepatic damage in non-steatotic grafts of the BD+anti-NRG1+IGF1+LT and steatotic grafts of the BD+anti-NRG1+VEGFA+LT were similar to those of the corresponding BD+anti-NRG1+LT group (Figure 2).

Given the results demonstrated above on the benefits of endogenous NRG1 in non-steatotic and steatotic LT from DBDs, we evaluated whether treatment with exogenous NRG1 might potentiate the benefits induced by endogenous NRG1 on hepatic injury, inflammation, and the regenerative response in both liver types. However, this was not the case. Treatment with exogenous NRG1 at a dose of 200 µg/kg (BD+NRG1(a)+LT group) resulted in parameters compatible with hepatic injury (transaminases, damage score, ALP, total bilirubin, vWF, and HA levels, Figure 2), inflammation (MDA, MPO, and edema, Figure 3), and cell proliferation (PCNA-positive hepatocytes, HGF, and TGF-β, Figure 5) similar to those in the BD+LT group. The ineffectiveness of exogenous NRG1 to alleviate hepatic damage, inflammation, and regenerative failure in non-steatotic and steatotic LT from DBDs was further confirmed by administering a higher dose of NRG1. When the dose was increased two-fold (BD+NRG1(b)+LT group), we observed more damage and regenerative failure in non-steatotic and steatotic liver grafts than observed in the BD+LT group (Figure 2, Figure 3 and Figure 5).

We also evaluated whether BD or obesity is contributing to the NRG1 increase. Our results indicated that, in steatotic and non-steatotic livers, NRG1 levels were similar in the Sham group (without any surgical intervention), the Prior to LT group (livers with cold ischemia but without BD), and the LT group (liver grafts with cold ischemia, without BD, and implanted in the recipient). However, the levels of hepatic NRG1 in the BD group (after BD induction but without cold ischemia) were increased as compared with the results of the Sham group. Such increases in hepatic NRG1 were also present in the Prior to BD+LT group (liver grafts after BD induction and undergoing cold ischemia). Interestingly, the levels of NRG1 in steatotic livers in all experimental groups analyzed were not different to those levels recorded in its respective analogous group carried out with non-steatotic livers (Supplementary Figure S1). These results indicate that BD is increasing NRG1 in the liver prior to LT, and that obesity is not related to increasing NRG1 in transplanted livers since NRG1 is not higher in steatotic livers prior to LT.

Once it was established that BD is the main event that induces an increase in NRG1 in the surgical setting of liver transplantation, we next evaluated what could be the source tissue of NRG1, and for this purpose we measured plasma and hepatic NRG1 levels at different time points after the induction of BD. Our results indicate that, in both types of livers, after BD induction, the increases in NRG1 are first observed in the circulation and later in the liver. In accordance with this, at 6 h after BD induction, we have observed increases in NRG1 in both circulation and the liver, whereas at early times (at 10 min or 1 h after the induction of BD), increases in NRG1 levels were only observed in the circulation but not in the liver (Supplementary Figure S1). These data point to the fact that in the first hours after the induction of BD, liver tissue is not the source of NRG1 but rather liver is taking up this mediator from the circulation.

As the BD event progresses, our results indicated that BD triggers a mechanistic process in both liver types to maintain elevated NRG1 levels, and this effect is observed in liver grafts from BD donors after cold ischemia (before the implantation of liver grafts in the recipient: the Prior to BD+LT group), as well as at 4 h after transplantation (after implantation of liver grafts: the BD+LT group). Moreover, it was observed that the increases in NRG1 are more evident in liver grafts from BD donors submitted to cold ischemia and before their implantation in the recipient (the Prior to BD+LT group), than in liver grafts after 6 h of BD induction and without cold ischemia (the BD group) (Supplementary Figure S1). In the conditions described for the Prior to BD+LT experimental group, the liver is isolated and is not affected by the influence of the circulation and other organs; therefore, such a finding indicates that the liver by itself is able to increase NGR1 at that time point of the liver transplantation process. This is also supported by the results observed in the BD+LT group (at 4 h after transplantation) because in this group NRG1 levels are increased in both circulation and the liver, in comparison with the levels observed in the Prior to BD+LT group. In the BD+LT group, the recipient of liver has not undergone BD, and for such a reason, circulating NRG1 is derived from the liver (Supplementary Figure S1). Hereby, NRG1 is increased in liver grafts that have suffered both the deleterious effects of BD and cold ischemia independently of the type of the liver. BD turn on signaling pathways in both liver types, which are then able to generate NRG1 and thereafter increase the levels for this mediator during several stages of the liver transplantation process.

### 3.2. Role of NRG1 in Steatotic and Non-Steatotic LT from DBDs in a Nutritionally Induced Obesity Model

Our results indicated that, similarly to the observed genetic obesity experimental model (Zucker rats) (Figure 2B), in the nutritional obesity model, an increase in ALP, bilirubin, vWF, and hyaluronic acid levels was evidenced in both steatotic and non-steatotic grafts of the BD+LT group when compared with the results of the LT group (Supplementary Figure S2). The inhibition of either NRG1 or PAK1 activity (BD+anti-NRG1+LT and BD+anti-PAK1+LT groups) increased ALP, bilirubin, vWF, and HA levels, in comparison with the BD+LT group, and in both liver types (Supplementary Figure S2). We administered VEGFA to Ln Wistar rats with non-steatotic livers after pharmacological inhibition of the NRG1-PAK1 pathway (BD+anti-NRG1+VEGFA+LT and BD+anti-PAK1+VEGFA+LT groups), as well as exogenous IGF1 to Ob Wistar rats with steatotic livers after pharmacological inhibition of the NRG1-PAK1 pathway (BD+anti-NRG1+IGF1+LT and BD+anti-PAK1+IGF1+LT groups). In these conditions, the levels of ALP, bilirubin, vWF, and HA were reduced, in comparison with experimental groups in which the effects of NRG1 or PAK1 were inhibited (BD+anti-NRG1+LT and BD+anti-PAK1+LT groups), thus resulting in similar parameters to those in the BD+LT group with steatotic and non-steatotic livers. The levels of ALP, bilirubin, vWF, and hyaluronic acid in non-steatotic grafts of the BD+anti-NRG1+IGF1+LT and steatotic grafts of the BD+anti-NRG1+VEGFA+LT were similar to those of the BD+LT group (Supplementary Figure S2).

## 4. Discussion

Although experimental and clinical studies indicate that plasma NRG1 levels are decreased in brain disorders such as schizophrenia or Parkinson’s disease [7,8], this seems to not be the case in BD conditions. Increased plasma and hepatic NRG1 levels were observed in LT from DBDs. We demonstrated, for the first time, the benefits of endogenous NRG1 in steatotic and non-steatotic LT from DBDs. BD triggers a mechanistic process in both liver types to maintain high levels of NRG1, which contribute to the mitigation of the damaging effects induced by BD. In fact, when endogenous NRG1 is pharmacologically inhibited, injury and inflammation are exacerbated, and failure in the proliferative response was detected. This investigation only focused on establishing whether there were BD-induced abnormalities in the levels of circulating NRG1 and, consequently, in the liver (and did not evaluate these levels in the brain). However, in light of the results obtained, it is possible to hypothesize that after BD induction, there is an increase in NRG1 in the brain that fosters a protective response against BD-induced brain injury, which would result in increased levels of NRG1 in the circulation and the liver. This has been observed in cases of brain injury due to trauma or cerebral ischemia, where there is an increase in the local expression of NRG1 in the brain, in an attempt to induce a protective response against brain injury [44,45,46,47]. Trauma leads to intracranial hypertension [48], which is one of the conditions present in our experimental model; in addition, the increase in intracranial pressure triggers secondary cerebral ischemia [49]. Therefore, in our experimental model of BD and considering that both conditions are present (intracranial hypertension and cerebral ischemia) [50,51], it is possible that an increase in the expression of NRG1 in the brain also occurs, as reflected in the high levels of NRG1 in the systemic circulation, and consequently in an increase in the hepatic levels of NRG1 as observed in our results.

In line with that described above, our results indicate that after BD induction, the increases in NRG1 are first observed in the circulation and later in the liver, thus indicating that in early hours after the start of the induction of BD, liver tissue is not the source of NRG1. During this time period, the main source of NRG1 is likely to be brain tissue, and NRG1 would be entering into liver from the circulation. As the BD event progresses, it engages molecular mechanisms in both liver types to maintain increased NRG1. In fact, after a period of hepatic ischemia an additional increase in NRG1 is induced during several stages of the liver transplantation process, as is observed in liver grafts from BD donors after cold ischemia, as well as after transplantation. In grafts from BD donors after cold ischemia (before transplantation), the liver is isolated and is not affected by the influence of the circulation and other organs; therefore, the liver by itself is able to increase NGR1 at that stage of the liver transplantation process. Regarding grafts undergoing BD, cold ischemia, and implantation in a recipient, NRG1 levels are even higher. Considering that the recipient of liver has not undergone BD, it could be concluded that, after transplantation circulation, NRG1 is derived from the liver. Accordingly, in the context of liver transplantation from DBD, brain death increases NRG1 in the liver prior to LT and as the liver transplant event progresses; hepatic cold ischemia also induces an additional increase in NRG1. Hepatic ischemia by itself (this means, without prior BD) is not able to increase NRG1. The present study has also demonstrated that obesity is not related to increasing NRG1 in transplanted livers since NRG1 is not different in steatotic and non-steatotic livers either prior to LT or after the implantation of liver grafts.

Although some literature shows that NRG1 interacts with leptin [52], this is not extrapolated to our experimental conditions. Indeed, our results indicate that endogenous and exogenous NRG1 can exert their functions with and without the presence of the leptin receptor since it can act similarly in homozygous Zucker rats (Ob) lacking the cerebral leptin receptor and in Ob Wistar rats (diet-induced) that have cerebral leptin receptors. Then, we conclude that NRG1 action is independent of the leptin receptor in this current model. In addition, the differences in the signaling pathway of NRG1 (NRG1-PAK1-VEGFA for non-steatotic livers and NRG1-PAK1-IGF1 for steatotic ones) are observed in genetic and nutritional obesity models used in the present study. Such different pathways are not surprising considering that there are different papers published in the literature supporting the differences in the molecular pathways depending on the type of the liver (steatotic vs. non-steatotic) [53,54,55,56,57]. Previously, research from our group has demonstrated that, in the setting of liver surgery, the presence or lack of leptin receptors in experimental models of liver steatosis does not influence the effects resulting from a therapeutic intervention. In this sense, a recent study [21] concluded that a different nutritional status equally affects steatotic livers from genetic obesity models and steatotic livers from nutritionally induced obesity in the context of liver transplantation. In addition, it has also been reported that nutritional status causes the same effects in both obesity models (genetic and nutritionally induced) in the context of partial hepatectomy surgery [58]. Moreover, the type of liver steatosis experimental model (from either nutritionally or genetic-induced obesity) did not affect the hepatic levels of the adipocytokines visfatin and resistin, observed in conditions of liver surgery [59].

Previous findings on the relationship between PAK1 and NRG1 have been mainly reported in breast cancer cells and tumor cell lines [12,13,14]. The originality of our results indicates that PAK-1 plays a role in the beneficial effects of NRG1 in liver grafts, establishing a signaling pathway in the setting of LT from DBDs that has not been previously described. In this study, we showed that the blockade of endogenous NRG1 diminished PAK1. In addition, and as occurred after NRG1 was inhibited, the abolishment of endogenous PAK1 activity led to an increase in the parameters of liver damage, inflammation, and regeneration, thus demonstrating a crucial role of this endogenous kinase limiting the poor postoperative outcomes in LT from DBDs. The involvement of PAK1 as a promoter of cell proliferation has been widely described in hepatocytes and hepatocellular carcinoma [15,60,61,62,63]. However, our results contradict the inflammatory role of PAK1 that has been described in aged liver and hepatocytes [64]; this might be explained, at least partially, by the important differences in the experimental models used (isolated hepatocytes in that study versus an in vivo LT surgical model in our research).

The NRG1-PAK1 pathway was responsible for modulating VEGFA levels in non-steatotic LT from DBDs. Pharmacological inhibition of the NRG1-PAK1 pathway in both types of livers decreased VEGFA only in the non-steatotic livers. In addition, blocking NRG1 or PAK1 in non-steatotic liver grafts from DBDs resulted in exacerbated injury, inflammation, and regenerative failure. This means that in non-steatotic liver grafts from DBDs, NRG1 and PAK1 are present; VEGFA is subsequently also induced; and the signaling pathway created by these three mediators attempts to mitigate liver damage, inflammation, and the regenerative failure that occurs in this type of surgical setting. If NRG1 or PAK1 fails, VEGFA expression is decreased and liver injury, inflammation, and regenerative failure are worsened. To reinforce this hypothesis on the development of the NRG1-PAK1-VEGFA pathway in non-steatotic LT from DBDs, we administered this pathway´s final effector, VEGFA, in non-steatotic grafts from DBDs whose NRG1 or PAK1 effects had been pharmacologically inhibited. We observed that treatment with exogenous VEGFA prevented deleterious effects in terms of injury, inflammation, and cell proliferation processes resulting from the blockade of either NRG1 or PAK1. Hence, this is the first study reporting that VEGFA is a pivotal mediator involved in the underlying beneficial effects of the endogenous NRG1-PAK1 pathway in non-steatotic LT from DBDs. Previous to this study, only one study demonstrated the involvement of the NRG1-PAK1-VEGFA pathway in angiogenesis in breast cancer epithelial cells [19].

A relationship between NRG1 and IGF1 or PAK1 and IGF1 has been demonstrated in different cell types [23,65,66,67,68]. To the best of our knowledge, the effect of NRG1-PAK1 on IGF1 had not been previously described. Our results indicated that in steatotic LT from DBDs, IGF1 was the downstream mediator of the NRG1-PAK1 pathway. When we inhibited the activity of either NRG1 or PAK1 in both types of liver grafts, a decrease in IGF-1 was only observed in steatotic organs. This event was associated with exacerbated damage, inflammation, and regenerative failure. In addition, exogenous IGF1 was administered in combination with antibodies to abolish NRG1 of PAK1 effects in steatotic grafts from DBDs. Under these conditions, injury, inflammation, and regenerative failure were mitigated in comparison with the results obtained when only the activity of either NRG1 or PAK1 was inhibited. Therefore, we herein demonstrated the existence of a new pathway, namely, NRG1-PAK1-IGF1, which benefits steatotic LT from DBDs. There are many structural and functional differences between hepatocytes with or without fatty infiltration [69,70,71], so it is not surprising that our results show a differential effect of the NRG1-PAK1 signaling pathway on VEGFA and IGF1, depending on the type of liver. In fact, there is vast evidence on the different signaling pathways underlying ischemia-reperfusion injury and regenerative failure in steatotic and non-steatotic livers [72,73,74].

Given the results mentioned above, the inhibition of endogenous NRG1 action exacerbated hepatic damage, inflammation, and the proliferative response failure in steatotic and non-steatotic LT from DBDs. Therefore, endogenous hepatic NRG1 protects both types of livers from the deleterious effects of BD. These results led us to evaluate whether the administration of exogenous NRG1 could be protective in liver grafts from deceased donors. However, we observed that when we administered exogenous NRG1 and combined with endogenous NRG1, injury and inflammation did not decrease, nor was there an improvement in liver regeneration markers with respect to the experimental group of BD+LT without treatment. This suggests that additional exogenous NRG1 did not protect against damage, inflammation, or regenerative failure in either type of liver graft. Even at high exogenous NRG1 doses, we observed these exacerbated deleterious effects. This is in contrast with data reported in numerous basic and clinical research studies indicating promising benefits from the administration of exogenous NRG1 in isolated hepatocytes, experimental models of cardiac regeneration or neurodegenerative diseases, and in heart-failure patients [8,75,76,77]; in steatotic and non-steatotic LT from DBDs, there were no observed benefits derived from the administration of NRG1. The results on exogenous NRG1 are of scientific and clinical interest since they provide new characteristics of this potential treatment and indicate that the administration of neurotrophic factors administered as a therapeutic option is not always beneficial. We demonstrated that exogenous NRG1 may not be appropriate for application in the clinical practice of LT from DBDs, independently of the type of liver (the presence or absence of steatosis). These findings could be highly relevant in a clinical scenario in which over 80% of all transplants are obtained from deceased donors and more than 50% of donors harbor hepatic steatosis. Clearly, intensive further investigations, which are outside of this study´s scope, will be necessary to determine whether these experimental results can be extrapolated to clinical practice and to completely exclude the use of exogenous NRG1 as a therapeutic strategy in clinical steatotic and non-steatotic LT from DBDs.

The differential effects of NRG1, regardless of its source (endogenous or exogenous), have been also observed with other mediators in the surgical setting of LT [54]. The deleterious effects of exogenous NRG1 could be due to either the dysregulation of the mediators involved in the cellular signaling downstream of the NRG1-PAK1-VEGFA/IGF1 pathway, or the harmful side effects derived from the administered drug. It is also possible that exogenous NRG1 could affect pathways other than those that are triggered by endogenous NRG1 (this reflects different mechanisms than the NRG1-PAK1-VEGFA/IGF1 pathway), and therefore, if such pathways are affected by exogenous NRG1, they could further promote and aggravate the observed detrimental effects. Interestingly, in conditions of hepatic ischemia-reperfusion injury, different mechanisms that underlie the effect of endogenous and exogenous sources of a mediator, for instance, nitric oxide, have been previously described [78]. Since exogenous NRG1 did not potentiate the endogenous protective activity of NRG1 in LT from DBD as described in this study, future research, of great scientific and clinical interest, could focus on different pathways such as NRG1 receptors or on signaling pathways upstream of NRG1, to finely regulate NRG1 and thus generate the appropriate levels required to decrease the incidence of postoperative complications in recipients of both steatotic and non-steatotic liver grafts obtained from deceased donors.

## 5. Conclusions

**Figure 6:**
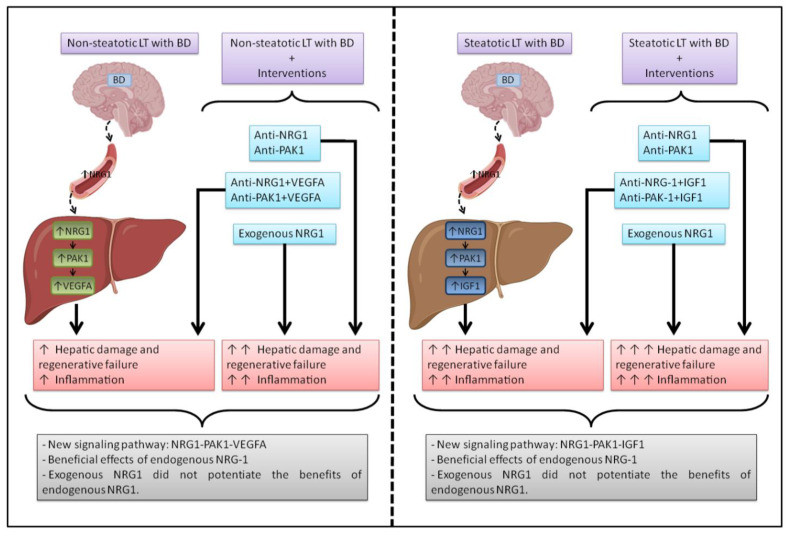
A schematic representation of the new proposed signaling pathways detected in this study, in steatotic and non-steatotic LT, and the effects of the different interventions and their outcomes.

In conclusion, this experimental study presents the different roles of endogenous and exogenous NRG1 and provides new mechanistic insights into endogenous NGR1 and its role in the pathology of LT from DBDs with steatotic and non-steatotic grafts. The results presented indicate that the baseline liver status (steatotic vs. non-steatotic) may ultimately dictate the signaling pathways by which endogenous NRG1 might exert its beneficial effects, that is, the NRG1-PAK1-VEGFA pathway in non-steatotic LT from DBDs and the NRG1-PAK1-IGF1 pathway in steatotic LT from DBDs (Figure 6). It is of clinical interest that this study modifies the dogma on the beneficial properties of therapies based on exogenous NRG1 administration because, in the conditions evaluated in this study, the use of exogenous NRG1 is not an appropriate strategy applicable to steatotic and non-steatotic LT from DBDs.

## Data Availability

The data presented in this study are available on request from the corresponding author.

## Supplementary Materials

The following supporting information can be downloaded at: https://www.mdpi.com/article/10.3390/biomedicines10050978/s1, Figure S1: Origin of NRG1 in LT from DBDs in a genetic obesity model; Figure S2: Relevance of NRG1 on hepatic functionality and cell endothelial damage in steatotic and non-steatoitc LT from DBDs in a nutritional obesity model.

## References

[1] Van Der Hoeven J.A., Moshage H., Schuurs T., Nijboer M., Van Schilfgaarde R., Ploeg R.J. (2003). Brain death induces apoptosis in donor liver of the rat. Transplantation.

[2] Kotsch K., Francuski M., Pascher A., Klemz R., Seifert M., Mittler J., Schumacher G., Buelow R., Volk H.D., Tullius S.G. (2006). Improved long-term graft survival after HO-1 induction in brain-dead donors. Am. J. Transpl..

[3] Reis-Júnior P., Tanigawa R., de Mesquita G.H.A., Basan N., Alves V., D’Albuquerque L.A.C., Andraus W. (2019). Steatosis and steatohepatitis found in adults after death due to non-burn trauma. Clinics.

[4] Croome K.P., Lee D.D., Taner C.B. (2019). The “Skinny” on Assessment and Utilization of Steatotic Liver Grafts: A Systematic Review. Liver Transpl..

[5] Heimbach J. (2014). Debate: A bridge too far-liver transplantation for nonalcoholic steatohepatitis will overwhelm the organ supply. Liver Transpl..

[6] Cespedes J.C., Liu M., Harbuzariu A., Nti A., Onyekaba J., Cespedes H.W., Bharti P.K., Solomon W., Anyaoha P., Krishna S. (2018). Neuregulin in Health and Disease. Int. J. Brain Disord. Treat..

[7] Wang R., Wang Y., Hu R., Chen X., Song M., Wang X. (2015). Decreased plasma levels of neureglin-1 in drug naïve patients and chronic patients with schizophrenia. Neurosci. Lett..

[8] Hama Y., Yabe I., Wakabayashi K., Kano T., Hirotani M., Iwakura Y., Utsumi J., Sasaki H. (2015). Level of plasma neuregulin-1 SMDF is reduced in patients with idiopathic Parkinson’s disease. Neurosci. Lett..

[9] Zhang P., Kuang H., He Y., Idiga S.O., Li S., Chen Z., Yang Z., Cai X., Zhang K., Potthoff M.J. (2018). NRG1-Fc improves metabolic health via dual hepatic and central action. JCI Insight.

[10] Meng D., Pan H., Chen Y., Ding J., Dai Y. (2021). Roles and mechanisms of NRG1 in modulating the pathogenesis of NAFLD through ErbB3 signaling in hepatocytes (NRG1 modulates NAFLD through ErbB3 signaling). Obes. Res. Clin. Pract..

[11] Dorsey W.C., Tchounwou P.B., Ford B.D. (2006). Neuregulin 1-Beta cytoprotective role in AML 12 mouse hepatocytes exposed to pentachlorophenol. Int. J. Environ. Res. Public Health.

[12] Maceyka M., Alvarez S.E., Milstien S., Spiegel S. (2008). Filamin A links sphingosine kinase 1 and sphingosine-1-phosphate receptor 1 at lamellipodia to orchestrate cell migration. Mol. Cell. Biol..

[13] Akinmade D., Talukder A.H., Zhang Y., Luo W.M., Kumar R., Hamburger A.W. (2008). Phosphorylation of the ErbB3 binding protein Ebp1 by p21-activated kinase 1 in breast cancer cells. Br. J. Cancer.

[14] Bourguignon L.Y., Gilad E., Peyrollier K. (2007). Heregulin-mediated ErbB2-ERK signaling activates hyaluronan synthases leading to CD44-dependent ovarian tumor cell growth and migration. J. Biol. Chem..

[15] Cao F., Yin L.X. (2020). PAK1 promotes proliferation, migration and invasion of hepatocellular carcinoma by facilitating EMT via directly up-regulating Snail. Genomics.

[16] Khare V., Dammann K., Asboth M., Krnjic A., Jambrich M., Gasche C. (2015). Overexpression of PAK1 promotes cell survival in inflammatory bowel diseases and colitis-associated cancer. Inflamm. Bowel Dis..

[17] Seo H.H., Lee S.Y., Lee C.Y., Kim R., Kim P., Oh S., Lee H., Lee M.Y., Kim J., Kim L.K. (2017). Exogenous miRNA-146a Enhances the Therapeutic Efficacy of Human Mesenchymal Stem Cells by Increasing Vascular Endothelial Growth Factor Secretion in the Ischemia/Reperfusion-Injured Heart. J. Vasc. Res..

[18] Xiao J., Li B., Zheng Z., Wang M., Peng J., Li Y., Li Z. (2012). Therapeutic effects of neuregulin-1 gene transduction in rats with myocardial infarction. Coron. Artery Dis..

[19] Bagheri-Yarmand R., Vadlamudi R.K., Wang R.A., Mendelsohn J., Kumar R. (2000). Vascular endothelial growth factor up-regulation via p21-activated kinase-1 signaling regulates heregulin-beta1-mediated angiogenesis. J. Biol. Chem..

[20] Bujaldon E., Cornide-Petronio M.E., Gulfo J., Rotondo F., Ávalos de León C., Negrete-Sánchez E., Gracia-Sancho J., Novials A., Jiménez-Castro M.B., Peralta Uroz C. (2019). Relevance of VEGFA in rat livers subjected to partial hepatectomy under ischemia-reperfusion. J. Mol. Med..

[21] Micó-Carnero M., Casillas-Ramírez A., Caballeria-Casals A., Rojano-Alfonso C., Sánchez-González A., Peralta C. (2021). Role of Dietary Nutritional Treatment on Hepatic and Intestinal Damage in Transplantation with Steatotic and Non-Steatotic Liver Grafts from Brain Dead Donors. Nutrients.

[22] Rupert C.E., Coulombe K.L.K. (2017). IGF1 and NRG1 Enhance Proliferation, Metabolic Maturity, and the Force-Frequency Response in hESC-Derived Engineered Cardiac Tissues. Stem Cells Int..

[23] Qiao M., Shapiro P., Kumar R., Passaniti A. (2004). Insulin-like growth factor-1 regulates endogenous RUNX2 activity in endothelial cells through a phosphatidylinositol 3-kinase/ERK-dependent and Akt-independent signaling pathway. J. Biol. Chem..

[24] Casillas-Ramírez A., Zaouali A., Padrissa-Altés S., Ben Mosbah I., Pertosa A., Alfany-Fernández I., Bintanel-Morcillo M., Xaus C., Rimola A., Rodés J. (2009). Insulin-like growth factor and epidermal growth factor treatment: New approaches to protecting steatotic livers against ischemia-reperfusion injury. Endocrinology.

[25] Zaouali M.A., Padrissa-Altés S., Ben Mosbah I., Alfany-Fernandez I., Massip-Salcedo M., Casillas-Ramirez A., Bintanel-Morcillo M., Boillot O., Serafin A., Rimola A. (2010). Improved rat steatotic and nonsteatotic liver preservation by the addition of epidermal growth factor and insulin-like growth factor-I to University of Wisconsin solution. Liver Transpl..

[26] Jiménez-Castro M.B., Meroño N., Mendes-Braz M., Gracia-Sancho J., Martínez-Carreres L., Cornide-Petronio M.E., Casillas-Ramirez A., Rodés J., Peralta C. (2015). The effect of brain death in rat steatotic and non-steatotic liver transplantation with previous ischemic preconditioning. J. Hepatol..

[27] Jiménez-Castro M.B., Negrete-Sánchez E., Casillas-Ramírez A., Gulfo J., Álvarez-Mercado A.I., Cornide-Petronio M.E., Gracia-Sancho J., Rodés J., Peralta C. (2017). The effect of cortisol in rat steatotic and non-steatotic liver transplantation from brain-dead donors. Clin. Sci..

[28] Kamada N., Calne R.Y. (1979). Orthotopic liver transplantation in the rat. Technique using cuff for portal vein anastomosis and biliary drainage. Transplantation.

[29] Van der Hoeven J.A., Lindell S., van Schilfgaarde R., Molema G., Ter Horst G.J., Southard J.H., Ploeg R.J. (2001). Donor brain death reduces survival after transplantation in rat livers preserved for 20 hours. Transplantation.

[30] Van Der Hoeven J.A., Ter Horst G.J., Molema G., de Vos P., Girbes A.R., Postema F., Freund R.L., Wiersema J., van Schilfgaarde R., Ploeg R.J. (2000). Effects of brain death and hemodynamic status on function and immunologic activation of the potential donor liver in the rat. Ann. Surg..

[31] Dominguez S.L., Hegde G.V., Hanson J.E., Xiang H., Mandikian D., Boswell C.A., Chiu C., Wu Y., Tsai S.P., Fleck D. (2018). Antibody-mediated stabilization of NRG1 induces behavioral and electrophysiological alterations in adult mice. Sci. Rep..

[32] Petrinovic M.M., Duncan C.S., Bourikas D., Weinman O., Montani L., Schroeter A., Maerki D., Sommer L., Stoeckli E.T., Schwab M.E. (2010). Neuronal Nogo-A regulates neurite fasciculation, branching and extension in the developing nervous system. Development.

[33] Jagadeeshan S., Subramanian A., Tentu S., Beesetti S., Singhal M., Raghavan S., Surabhi R.P., Mavuluri J., Bhoopalan H., Biswal J. (2016). P21-activated kinase 1 (Pak1) signaling influences therapeutic outcome in pancreatic cancer. Ann. Oncol..

[34] Bockhorn M., Goralski M., Prokofiev D., Dammann P., Grünewald P., Trippler M., Biglarnia A., Kamler M., Niehues E.M., Frilling A. (2007). VEGF is important for early liver regeneration after partial hepatectomy. J. Surg. Res..

[35] Gu Y., Sowa J.P., Paul A., Gerken G., Schlaak J.F. (2013). Vascular endothelial growth factor improves liver regeneration and survival after 90% hepatectomy in a rat model of diet-induced steatosis. Digestion.

[36] Ennequin G., Boisseau N., Caillaud K., Chavanelle V., Etienne M., Li X., Sirvent P. (2015). Neuregulin 1 Improves Glucose Tolerance in db/db Mice. PLoS ONE.

[37] Serafín A., Roselló-Catafau J., Prats N., Xaus C., Gelpí E., Peralta C. (2002). Ischemic preconditioning increases the tolerance of Fatty liver to hepatic ischemia-reperfusion injury in the rat. Am. J. Pathol..

[38] Peralta C., Prats N., Xaus C., Gelpí E., Roselló-Catafau J. (1999). Protective effect of liver ischemic preconditioning on liver and lung injury induced by hepatic ischemia-reperfusion in the rat. Hepatology.

[39] Wang R.H., Li C., Deng C.X. (2010). Liver steatosis and increased ChREBP expression in mice carrying a liver specific SIRT1 null mutation under a normal feeding condition. Int. J. Biol. Sci..

[40] Riva G., Villanova M., Cima L., Ghimenton C., Bronzoni C., Colombari R., Crestani M., Sina S., Brunelli M., D’Errico A. (2018). Oil Red O Is a Useful Tool to Assess Donor Liver Steatosis on Frozen Sections during Transplantation. Transpl. Proc..

[41] Franco-Gou R., Peralta C., Massip-Salcedo M., Xaus C., Serafín A., Roselló-Catafau J. (2004). Protection of reduced-size liver for transplantation. Am. J. Transpl..

[42] Burr A.W., Toole K., Chapman C., Hines J.E., Burt A.D. (1998). Anti-hepatocyte growth factor antibody inhibits hepatocyte proliferation during liver regeneration. J. Pathol..

[43] Russell W.E., Coffey R.J., Ouellette A.J., Moses H.L. (1988). Type beta transforming growth factor reversibly inhibits the early proliferative response to partial hepatectomy in the rat. Proc. Natl. Acad. Sci. USA.

[44] Deng W., Luo F., Li B.M., Mei L. (2019). NRG1-ErbB4 signaling promotes functional recovery in a murine model of traumatic brain injury via regulation of GABA release. Exp. Brain Res..

[45] Parker M.W., Chen Y., Hallenbeck J.M., Ford B.D. (2002). Neuregulin expression after focal stroke in the rat. Neurosci. Lett..

[46] Tokita Y., Keino H., Matsui F., Aono S., Ishiguro H., Higashiyama S., Oohira A. (2001). Regulation of neuregulin expression in the injured rat brain and cultured astrocytes. J. Neurosci..

[47] Guo W.P., Wang J., Li R.X., Peng Y.W. (2006). Neuroprotective effects of neuregulin-1 in rat models of focal cerebral ischemia. Brain Res..

[48] Stroh J.N., Bennett T.D., Kheyfets V., Albers D. (2021). Clinical Decision Support for Traumatic Brain Injury: Identifying a Framework for Practical Model-Based Intracranial Pressure Estimation at Multihour Timescales. JMIR Med. Inform..

[49] Hlatky R., Valadka A.B., Robertson C.S. (2003). Intracranial hypertension and cerebral ischemia after severe traumatic brain injury. Neurosurg. Focus.

[50] Cacciatori A., Godino M., Mizraji R. (2018). Utility of Transcranial Doppler in the Coordination of Transplants: 10 Years of Experience. Transpl. Proc..

[51] Harrer J.U., Eyding J., Ritter M., Schminke U., Schulte-Altedorneburg G., Köhrmann M., Nedelmann M., Schlachetzki F. (2012). The potential of neurosonography in neurological emergency and intensive care medicine: Monitoring of increased intracranial pressure, brain death diagnostics, and cerebral autoregulation—Part 2. Ultraschall Med..

[52] Ennequin G., Boisseau N., Caillaud K., Chavanelle V., Etienne M., Li X., Montaurier C., Sirvent P. (2015). Neuregulin 1 affects leptin levels, food intake and weight gain in normal-weight, but not obese, db/db mice. Diabetes Metab..

[53] Selzner M., Rüdiger H.A., Sindram D., Madden J., Clavien P.A. (2000). Mechanisms of ischemic injury are different in the steatotic and normal rat liver. Hepatology.

[54] Carrasco-Chaumel E., Roselló-Catafau J., Bartrons R., Franco-Gou R., Xaus C., Casillas A., Gelpí E., Rodés J., Peralta C. (2005). Adenosine monophosphate-activated protein kinase and nitric oxide in rat steatotic liver transplantation. J. Hepatol..

[55] Casillas-Ramírez A., Ben Mosbah I., Ramalho F., Roselló-Catafau J., Peralta C. (2006). Past and future approaches to ischemia-reperfusion lesion associated with liver transplantation. Life Sci..

[56] Casillas-Ramirez A., Amine-Zaouali M., Massip-Salcedo M., Padrissa-Altés S., Bintanel-Morcillo M., Ramalho F., Serafín A., Rimola A., Arroyo V., Rodés J. (2008). Inhibition of angiotensin II action protects rat steatotic livers against ischemia-reperfusion injury. Crit. Care Med..

[57] Álvarez-Mercado A.I., Negrete-Sánchez E., Gulfo J., Ávalos de León C.G., Casillas-Ramírez A., Cornide-Petronio M.E., Bujaldon E., Rotondo F., Gracia-Sancho J., Jiménez-Castro M.B. (2019). EGF-GH Axis in Rat Steatotic and Non-steatotic Liver Transplantation from Brain-dead Donors. Transplantation.

[58] Mendes-Braz M., Elias-Miró M., Kleuser B., Fayyaz S., Jiménez-Castro M.B., Massip-Salcedo M., Gracia-Sancho J., Ramalho F.S., Rodes J., Peralta C. (2014). The effects of glucose and lipids in steatotic and non-steatotic livers in conditions of partial hepatectomy under ischaemia-reperfusion. Liver Int..

[59] Elias-Miró M., Mendes-Braz M., Cereijo R., Villarroya F., Jiménez-Castro M.B., Gracia-Sancho J., Guixé-Muntet S., Massip-Salcedo M., Domingo J.C., Bermudo R. (2014). Resistin and visfatin in steatotic and non-steatotic livers in the setting of partial hepatectomy under ischemia-reperfusion. J. Hepatol..

[60] Zhang Z.L., Liu G.C., Peng L., Zhang C., Jia Y.M., Yang W.H., Mao L. (2018). Effect of PAK1 gene silencing on proliferation and apoptosis in hepatocellular carcinoma cell lines MHCC97-H and HepG2 and cells in xenograft tumor. Gene Ther..

[61] Iyer S.C., Gopal A., Halagowder D. (2015). Myricetin induces apoptosis by inhibiting P21 activated kinase 1 (PAK1) signaling cascade in hepatocellular carcinoma. Mol. Cell. Biochem..

[62] Wong L.L., Lam I.P., Wong T.Y., Lai W.L., Liu H.F., Yeung L.L., Ching Y.P. (2013). IPA-3 inhibits the growth of liver cancer cells by suppressing PAK1 and NF-κB activation. PLoS ONE.

[63] Parekh P., Motiwale L., Naik N., Rao K.V. (2011). Downregulation of cyclin D1 is associated with decreased levels of p38 MAP kinases, Akt/PKB and Pak1 during chemopreventive effects of resveratrol in liver cancer cells. Exp. Toxicol. Pathol..

[64] Kim D.H., Park M.H., Chung K.W., Kim M.J., Park D., Lee B., Lee E.K., Choi Y.J., Kim N.D., Yu B.P. (2015). Suppression of FoxO6 by lipopolysaccharide in aged rat liver. Oncotarget.

[65] Kennedy L.M., Pham S.C., Grishok A. (2013). Nonautonomous regulation of neuronal migration by insulin signaling, DAF-16/FOXO, and PAK-1. Cell Rep..

[66] Kehat I., Molkentin J.D. (2010). Molecular pathways underlying cardiac remodeling during pathophysiological stimulation. Circulation.

[67] Worthington J., Bertani M., Chan H.L., Gerrits B., Timms J.F. (2010). Transcriptional profiling of ErbB signalling in mammary luminal epithelial cells—interplay of ErbB and IGF1 signalling through IGFBP3 regulation. BMC Cancer.

[68] Takahashi K., Tanaka T., Suzuki K. (2010). Directional control of WAVE2 membrane targeting by EB1 and phosphatidylinositol 3,4,5-triphosphate. Cell. Signal..

[69] Angulo P. (2002). Nonalcoholic fatty liver disease. New. Engl. J. Med..

[70] Veteläinen R., van Vliet A., Gouma D.J., van Gulik T.M. (2007). Steatosis as a risk factor in liver surgery. Ann. Surg..

[71] Farrell G.C., Teoh N.C., McCuskey R.S. (2008). Hepatic microcirculation in fatty liver disease. Anat. Rec..

[72] Peralta C., Jiménez-Castro M.B., Gracia-Sancho J. (2013). Hepatic ischemia and reperfusion injury: Effects on the liver sinusoidal milieu. J. Hepatol..

[73] Selzner M., Clavien P.A. (2001). Fatty liver in liver transplantation and surgery. Semin. Liver Dis..

[74] Núñez K., Thevenot P., Alfadhli A., Cohen A. (2018). Complement Activation in Liver Transplantation: Role of Donor Macrosteatosis and Implications in Delayed Graft Function. Int. J. Mol. Sci..

[75] Ganapathy B., Nandhagopal N., Polizzotti B.D., Bennett D., Asan A., Wu Y., Kühn B. (2016). Neuregulin-1 Administration Protocols Sufficient for Stimulating Cardiac Regeneration in Young Mice do not Induce Somatic, Organ, or Neoplastic Growth. PLoS ONE.

[76] Jalilzad M., Jafari A., Babaei P. (2019). Neuregulin1β improves both spatial and associative learning and memory in Alzheimer model of rats possibly through signaling pathways other than Erk1/2. Neuropeptides.

[77] Lenihan D.J., Anderson S.A., Lenneman C.G., Brittain E., Muldowney J.A.S., Mendes L., Zhao P.Z., Iaci J., Frohwein S., Zolty R. (2016). A Phase I, Single Ascending Dose Study of Cimaglermin Alfa (Neuregulin 1β3) in Patients with Systolic Dysfunction and Heart Failure. JACC Basic Transl. Sci..

[78] Peralta C., Rull R., Rimola A., Deulofeu R., Roselló-Catafau J., Gelpí E., Rodés J. (2001). Endogenous nitric oxide and exogenous nitric oxide supplementation in hepatic ischemia-reperfusion injury in the rat. Transplantation.

