# Supplementary material for: The Role of Neuregulin-1 in Steatotic and Non-Steatotic Liver Transplantation from Brain-Dead Donors
Source: Biomedicines. 2022 Apr 23;10(5):978. doi: 10.3390/biomedicines10050978 (PMC9138382; doi:10.3390/biomedicines10050978)
Supplement: Supplementary file 1 [file biomedicines-10-00978-s001.zip › biomedicines-1640858-supplementary.pdf]

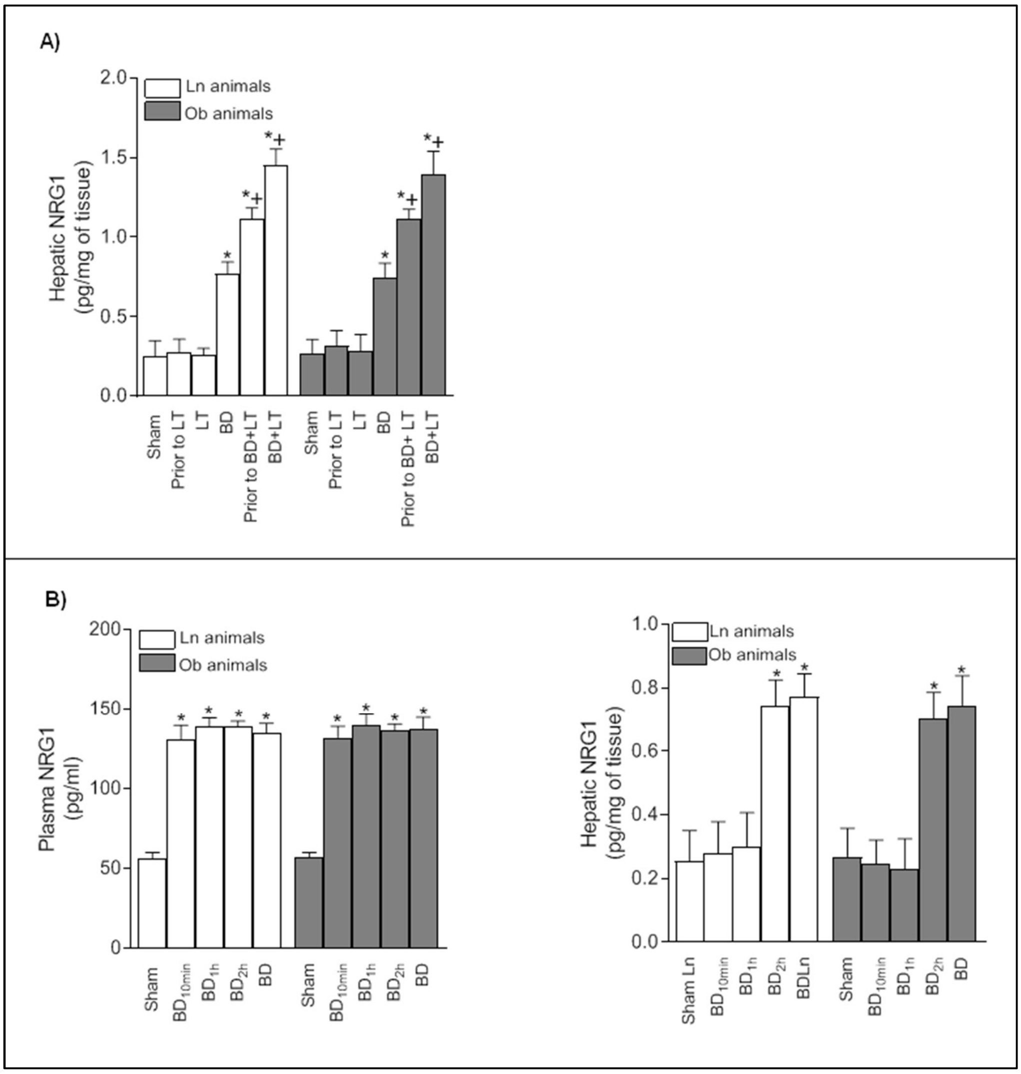

Supplementary Figure S1. Origin of NRG1 in LT from DBDs in a genetic obesity model. (A) Levels of NRG1 In steatotic and non-steatotic livers. B) NRG1 levels in plasma and liver at 10 min, 2 and 6 hours after the BD induction. *P < 0.05 versus Sham; +P < 0.05 versus LT.

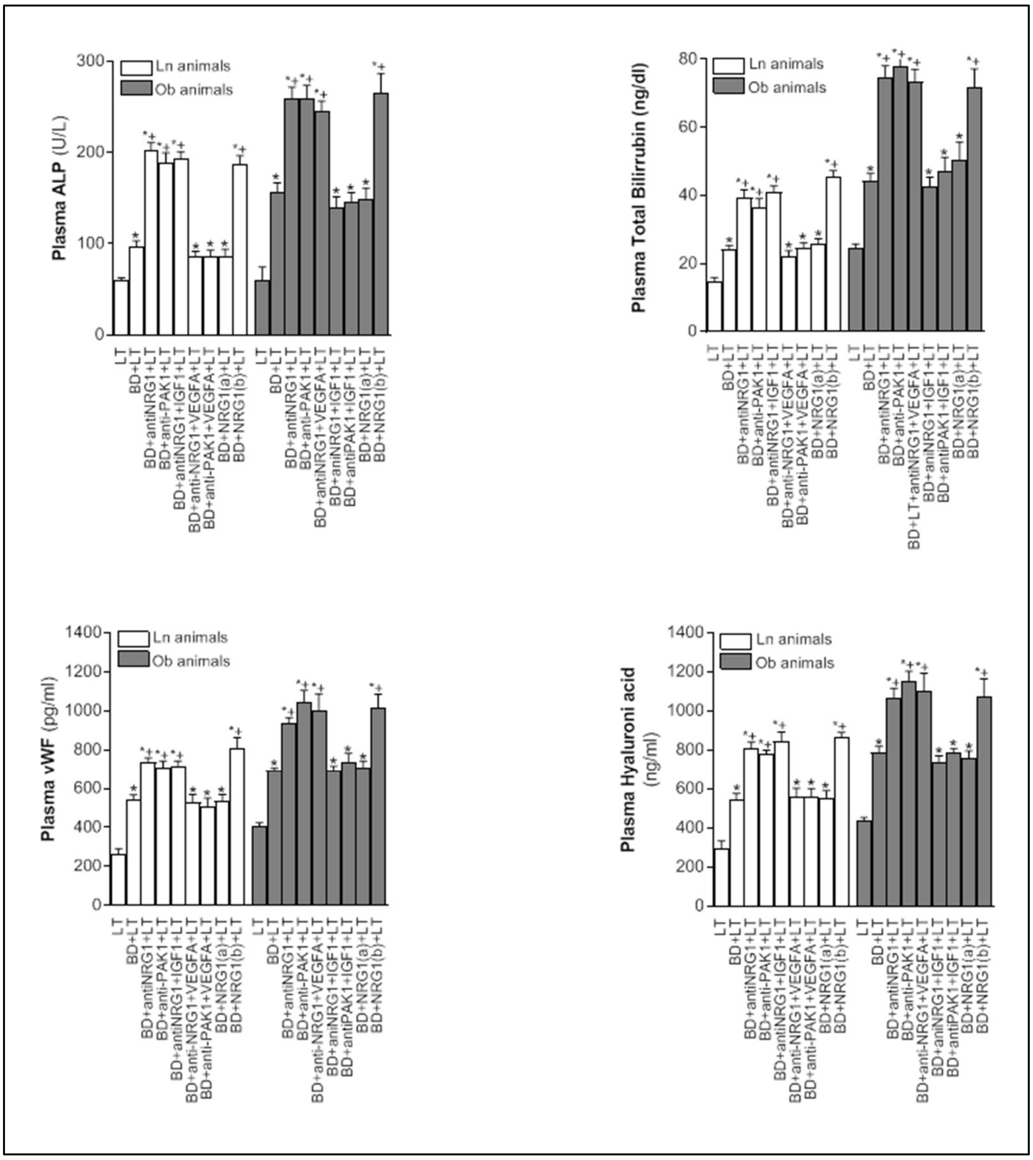

Supplementary Figure S2. Relevance of NRG1 on hepatic functionality and cell endothelial damage in steatotic and non-steatoitc LT from DBDs in a nutritional obesity model. ALP, total bilirubin levels, vWF, and HA levels in plasma. *P < 0.05 versus LT; +P < 0.05 versus BD+LT.
